# Epidemiology of Injuries and Their Implications in Jiu-Jitsu Practitioners: An Integrative Systematic Review

**DOI:** 10.1055/s-0044-1785662

**Published:** 2024-06-22

**Authors:** Sara Pereira Santos, Higor Henrique Pinheiro Soares, Sebastião Perez Neto, Luis Carlos Caseiro Filho, Carlos Eduardo Girasol

**Affiliations:** 1 Departamento de Fisioterapia, Centro Universitário Estácio de Ribeirão Preto, Ribeirão Preto, SP, Brasil; 2 Departamento de Ciências da Saúde, Faculdade de Medicina de Ribeirão Preto, Universidade de São Paulo (FMRP-USP), Ribeirão Preto, SP, Brasil

**Keywords:** cumulative trauma disorders, martial arts, sports, sports medicine

## Abstract

**Objective** To investigate the epidemiology of injury types among jiu-jitsu practitioners, as well as the incidence regarding different skill and experience levels, through the question: “What are the characteristics and prevalence of musculoskeletal injuries in Jiu-Jitsu practitioners?”.

**Methods** Since the beginning of the study, in August 2020, we conducted a search on the MEDLINE, LILACS, and SciELO electronic databases. We included cross-sectional studies published between 2018 and 2023 on the epidemiology of the types of injuries among jiu-jitsu practitioners that compared their incidence regarding different levels of ability and experience. Two independent researchers performed the data extraction and assessed the risk of bias.

**Results** Seven studies were included. The common outcomes involved 2,847 jiu-jitsu practitioners. A high prevalence in the knee joint and chest and rib areas was reported. Considering the difference in experience level among the practitioners, we could observe that most of the individuals included were beginners. Among the age groups observed, male practitioners older than 30 years of age were the ones who presented the highest rate of musculoskeletal injury, especially during training sessions.

**Conclusion** There is a high prevalence of musculoskeletal injuries among jiu-jitsu practitioners. The most affected anatomical segments are the knee joint, the chest, and the rib region, followed by the shoulder joint. The related factors change according to certain variables, being more common during training in male individuals over 30 years of age and beginners in the sport.

## Introduction

The fights in combat sports can result in soft tissue injuries 1 and even structural trauma; 2 these injuries may be severe and require the performance of arthroplasty in certain structures, 3 and they can also lead to an interruption of the fight or withdrawal from the sport.

Jiu-jitsu is recognized as a combat sport in which athletes use “finishing” techniques on the opponent and many times the outcome of such techniques is a joint block, termed “submission hold” by the practitioners. 4 The fight takes place with unexpected, fast, repetitive, and high-overload movements for muscles and joints. The association of these factors with high-volume training can favor the appearance of musculoskeletal and osteoarticular injuries. 5

In this martial art, the high physical impact and exposure to daily training sessions corroborate the outcomes observed in the literature, with a higher incidence of injuries during training when compared to competitions. 6 7 8 9

Some authors emphasize that the incidence of injury is very similar among martial arts. However, there are few studies associated with injuries arising specifically from the practice of jiu-jitsu. 4 5 10 11 Since injuries are characterized according to severity, treatment time, nature of the injury, and intrinsic characteristics, 4 12 knowledge of the incidence and prevalence of orthopedic injuries, as well as of their characteristics, yields direct benefits for the treatment, prevention, and promotion of strategies for such events. Thus, the objective of the present study was to investigate the epidemiology of injury types among jiu-jitsu practitioners, as well as the incidence at different skill and experience levels, through the question “What are the characteristics and prevalence of musculoskeletal injuries in jiu-jitsu practitioners?”. The present systematic review provides a synthesis and evidence for the scientific and clinical communities concerning these conditions and possible healthcare support for jiu-jitsu practitioners.

## Materials and Methods

### Search Strategy

The present systematic review was conducted according to the Preferred Reporting Items for Systematic Reviews and Meta-Analyses (PRISMA) statement. 13 14 The study protocol was prepared and registered on the Prospective Register of Systematic Reviews (PROSPERO) under identification CRD42023422767. The search was carried out on the MEDLINE (PubMed), LILACS, and SciELO electronic databases, and the strategy used was the combination and adaptation of Medical Subject Headings (MeSH) of the National Library of Medicine: *jiu-jitsu* , *Brazilian jiu-jitsu* , and *injuries* ( Table 1 ). The search was carried out from the inception of the study up to May 2023. The lists of bibliographic references were revised to identify other potential studies.

**Table 1:** Search strategies

Search	Query
#1	( *prevalence [MeSH Terms]* OR *prevalence* OR *prevalences* OR *occurrence* OR *frequency* OR *frequencies* OR *incidence* OR *epidemiology* OR *epidemiologic* OR *incidence* OR *incidences* )
#2	( *injuries* OR *injury* OR *sports injuries* OR *sports injury* OR *injuries, sports* OR *injury, sports* OR *injuries, athletic* OR *athletic injury* OR *injury, athletic* )
#3	( *jiujitsu* OR *jiu-jitsu* OR *jujitsu* OR *Brazilian jiujitsu* OR *Brazilian jiu-jitsu* )
#4	(#1 AND #2 AND #3)

The Population, Intervention, Comparison, Outcomes, Studies (PICOS) strategy was used to formulate the question in the present systematic review, in which P represents the jiu-jitsu practitioners, I and C are not applicable, O is the prevalence of musculoskeletal injuries and the most frequent types among jiu-jitsu practitioners, and S represents the observational studies. No intervention or comparison were performed in the present systematic review.

### Study Selection

The studies identified in the search were entered into a standard Excel spreadsheet (Microsoft Corp., Redmond, WA, United States) for the exclusion of duplicates. Two independent researchers (HHPS and SPN) employed the selection strategy based on the contents of the title and abstract. Subsequently, the full texts of the studies were retrieved for eligibility assessment. The inclusion criteria were cross-sectional studies involving jiu-jitsu practitioners and musculoskeletal injuries. Studies published in languages other than English or Portuguese, as well as incomplete texts, were excluded. In case of disagreement between researchers, resolution was achieved through discussions. In the case of persistence of any disagreement, the evaluation of a third researcher (CEG) was requested.

### Data Extraction and Assessment of the Risk of Bias

Two independent researchers (HHPS and SPN) performed data extraction and assessed the risk of bias. A predefined form was used for data collection, encompassing the first author, the year of publication, the practitioners' level, age, gender, the number of athletes, the training frequency, the anatomical region of the musculoskeletal injury, and the moment of injury onset. The risk of bias in observational studies of exposure was assessed using the Cochrane Collaboration's Risk of Bias In Non-Randomized Studies – of Exposure (ROBINS-E) 15 tool, which encompasses seven methodological domains: confounding, measurement of the exposure, selection of participants, postexposure interventions, missing data, measurement of outcomes, and selection of the reported result. The score is established as low (-), high (+), and uncertain (?) risk of bias.

## Results

**Fig. 1:**
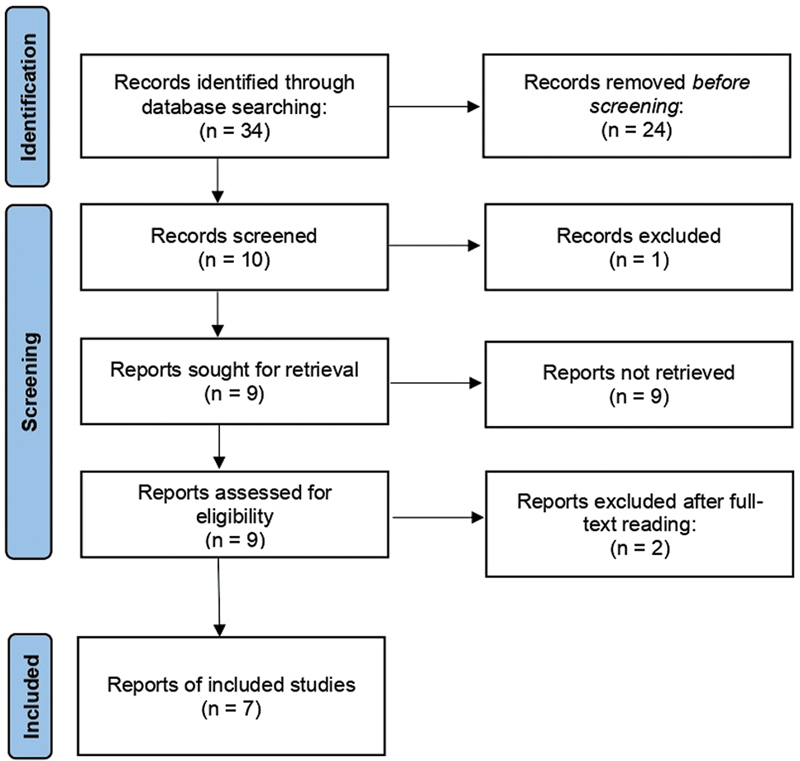
The Preferred Reporting Items for Systematic Reviews and Meta-Analyses (PRISMA) statement flow diagram of the study selection process.

Following a systematic literature search, a total of 34 articles were found on the main electronic databases. No studies were selected from the grey literature, since the identified records were already on the other databases. After duplicates were excluded and title and abstract screening was concluded, ten records remained. Subsequently, one record was discarded, and nine studies were considered eligible to be fully assessed. After full-text reading, seven studies 4 7 8 9 16 17 18 were finally included for qualitative and quantitative synthesis. An overview of the selection process is shown in Fig. 1 .

### Study Characteristics

The cross-sectional studies included in the present systematic review, 4 7 8 9 16 17 18 published between 2018 and 2023, totaled 2,847 jiu-jitsu practitioners. Table 2 shows the characteristics of the articles included.

**Table 2:** Characteristics of studies that fulfilled the inclusion criteria

Authors and year of publication		Country	n	Objectives	Data collection
da Silva Junior et al., 16 2018		Brazil	108	To verify the regions of the body affected by injuries, the most common site of injury, and the mechanism and severity of injuries in beginners and advanced jiu-jitsu practitioners.	Questionnaire
Lopes et al., 17 2018		Brazil	31	To study possible relations between the prevalence of injuries and the functional system of movement of jiu-jitsu fighters.	Questionnaire
Petrisor et al., 18 2019		Canada	70	To describe injuries experienced during jiu-jitsu training, both in practice and competition, and to classify the type of injury and explore the characteristics of the participant and the injury associated with the desire to quit jiu-jitsu after the injury.	Questionnaire
Moriarty et al., 9 2019		USA	1,287	To determine the six-month incidence rate and related jiu-jitsu pattern injuries and to characterize associations between injuries and experience level, demographic factors, and training variables.	Questionnaire
Nicolini et al., 4 2021		Brazil	96	To identify an epidemiological profile of orthopedic injuries present in jiu-jitsu practitioners.	Questionnaire
Hinz et al., 7 2021		Germany	1,140	To quantify the incidence of related injuries in jiu-jitsu over three years and to detect common injury patterns and risk factors among jiu-jitsu practitioners.	Questionnaire
Nery et al., 8 2023		Brazil	115	To determine the prevalence of musculoskeletal injuries in jiu-jitsu competitors, as well as their profile and characteristics.	Questionnaire

### Definition of Musculoskeletal Injury in Sport

Musculoskeletal injuries are common and account for a significant burden to the healthcare system. According to Caine et al., 19 Hoff and Martin, 20 and Van Mechelen et al., 21 sports injuries are the collective name to characterize all types of damages related to physical activities. Corroborating the authors' discussion, Timpka et al. 22 define them etiologically as a loss of body functions or deviation of structure caused by the transfer of energy during participation in sport. Therefore, one can distinguish between acute injuries, which occur by interaction with a relatively high force in a short time of stimulus, and chronic injuries, which result from the effect of the force applied in repetition, even in lower intensity, such as overuse. 23 24 Thus, based on this definition, the present systematic review aims to provide understanding about these results reported in the literature.

### Injury Epidemiology: Structure, Age, and Timing of Injury

**Fig. 2:**
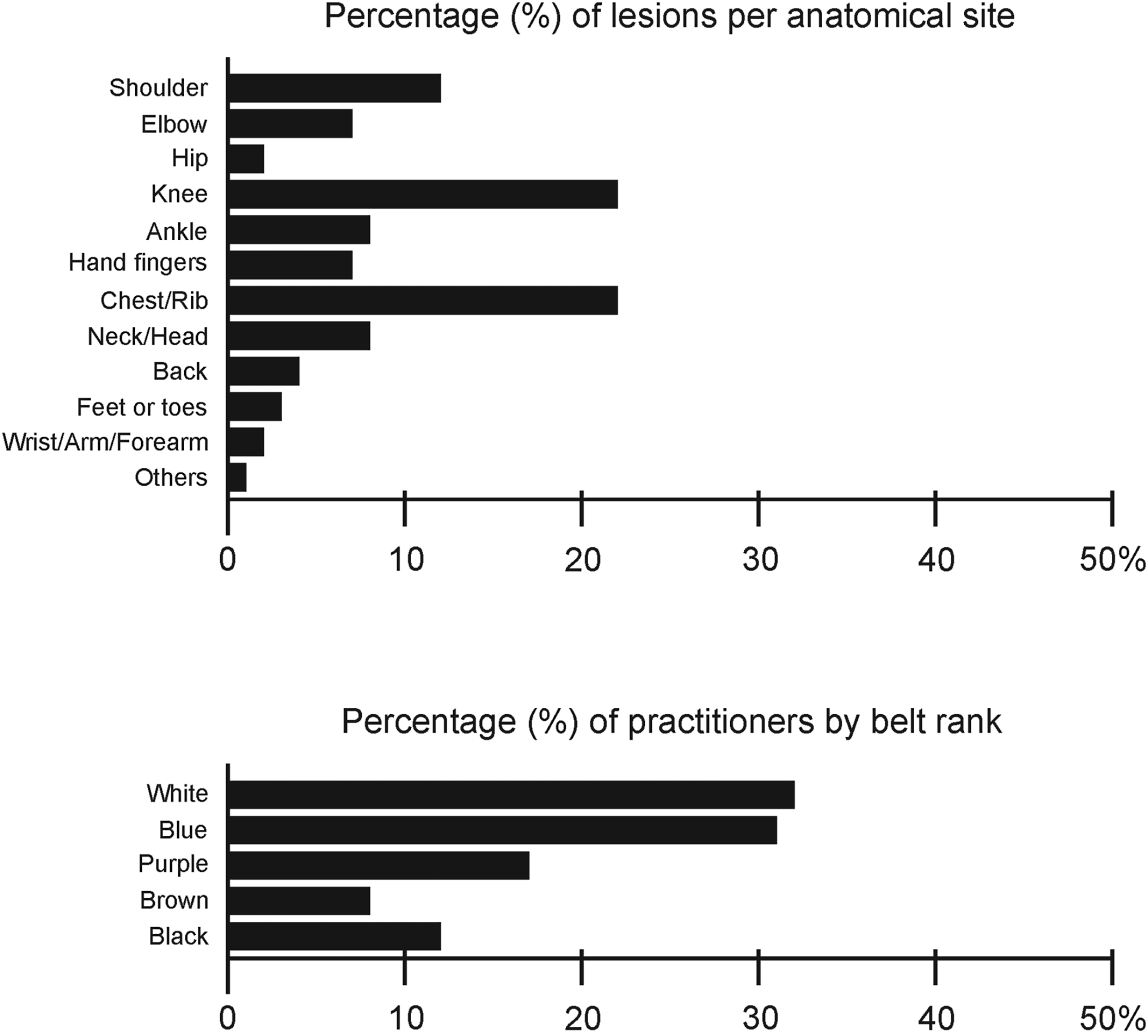
Epidemiological characteristics of the injuries and level of experience of jiu-jitsu practitioners.

The distribution of the occurrence of musculoskeletal injuries according to the included studies 4 7 8 9 16 17 18 shows the high prevalence in the knee joint (22%) and chest and rib regions (22%), with rates close to half of the total cases reported in the present study. Considering the difference in the level of experience among the practitioners, it is possible to observe that most subjects included were white and blue belts, that is, beginners in the sport. The total distribution of the prevalence of injuries and experience of the practitioners can be seen in Fig. 2 .

Considering age groups, participants older than 18 years of age were included, which hampers the analysis of data from younger practitioners. In this regard, according to the data available, it is noticeable that male practitioners older than 30 years of age are more susceptible to experiencing a musculoskeletal injury. Another remarkable characteristic is the high rate of occurrence of injuries during training sessions compared to competitions. The complete data can be seen in Table 3 .

**Table 3:** Epidemiology of injury characteristics

Study	Age (years)			Frequency of injuries (%)			Time of Injury	
	Mean(±standard deviation)	Inclusion	Highest frequency	Men	Women	Total	Competition	Training
da Silva Junior et al. 16	28.3(±6.41)	Not reported	Not reported	Not reported	Not reported	100%	0%	100%
Lopes et al. 17	30.9(±7.3)	Not reported	≥ 30	100%	Not reported	100%	0%	100%
Petrisor et al. 18	–	> 18	≥ 30	Not reported	Not reported	91.4%	8.6%	100%
Moriarty et al. 9	29.5(±2.12)	> 18	26 to 35	15.5%	84.5%	59%	2%	98%
Nicolini et al. 4	27.6(±1.42)	18 to 45	Not reported	86%	84%	84%	16%	100%
Hinz et al. 7	31.7(±7.9)	Not reported	32.12	89.7	10.2%	48.7%	51.30%	48.70%
Nery et al. 8	25.8(±4.1)	Not reported	Not reported	Not reported	Not reported	85.2%	41%	59%

### Risk of Bias Assessment

**Fig. 3:**
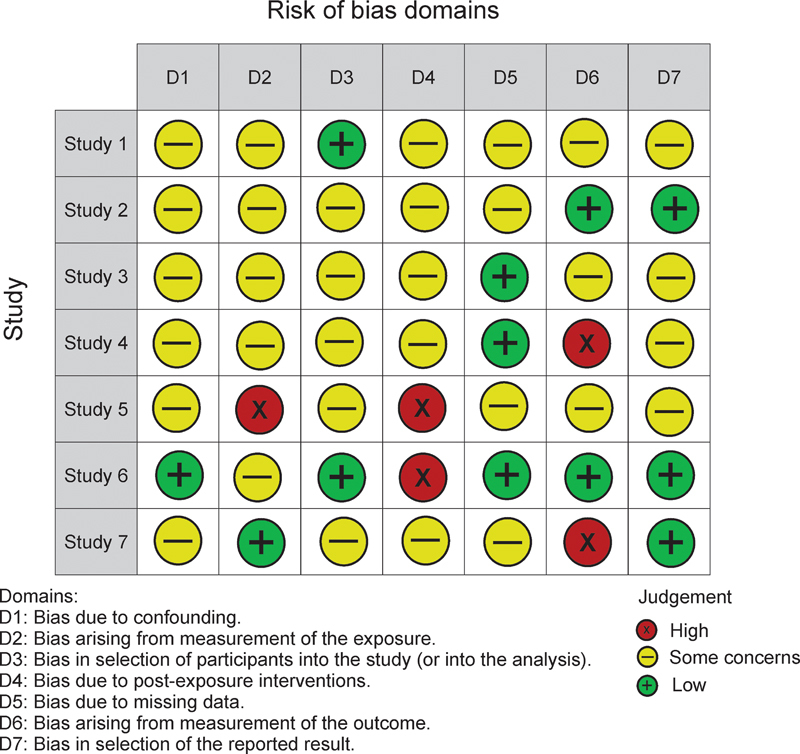
Summary of authors' review judgments about each risk of bias item for each included study.

Overall, there was a moderate risk of bias for the studies included 4 7 8 9 16 17 18 ( Fig. 3 ). The descriptions of the protocols and methods addressed were not appropriate for a full interpretation of confounders and outcome measurements. Participation, random sequence, and allocation concealment were robustly identified in three studies. 4 8 9 In general, the studies did not add much emphasis to data loss or erroneous exposure, as well as to outcome measurement bias. Fig. 3 presents the assessment of each risk of bias item for the studies included in the present systematic review.

## Discussion

The present study is a systematic review of the literature on the prevalence of musculoskeletal injuries in jiu-jitsu practitioners, evaluating the relationship regarding the most affected anatomical segments, age, gender, belt, and time of the injury. Therefore, we could highlight the anatomical regions with a higher incidence of injury that should be the most concerning for jiu-jitsu practitioners and instructors.

It is important to underline that martial arts are based on biomechanical principles that can cause severe damage to practitioners. 5 25 Due to the fighting technique, which exposes practitioners to falls and shocks with a great application of force, orthopedic injuries are very common in the practice of the sport. Approximately 90% of the participating athletes reported the occurrence of at least one injury. 26 However, when examining the incidence of these injuries according to demographic variables, we were unable to find a consistent factor that had a significant impact on their occurrence.

In the present systematic review, a higher prevalence of injuries to the knee, chest, and ankle was found when compared to other anatomical segments. This situation also applies when we evaluate studies that involved other martial arts, such as judo. 27 Furthermore, as seen in Pocecco et al., 28 the fingers and hands are the most affected sites of injury in judo, which differs from jiu-jitsu, as it can be seen in the data of the present study. In other modalities, such as mixed martial arts, which allow more acute and incisive contact, such as punching and kicking, the prevalence of injuries is higher in the head and neck. In these modalities, lacerations, abrasions, and contusions are the most frequent, besides conditions such as concussion.

Through our research, we could notice the low number of studies on the subject, which makes it even more difficult to interpret the results. Factors such as the lack of standardization in the classification of athletes as beginners or experienced also hindered inferences about the results. To illustrate this, da Silva Junior et al. 16 considered beginners the athletes who were white or blue belts, while Petrisor et al. 18 considered only white-belt athletes as beginners, while athletes of other belts were considered advanced. It would be interesting if future studies standardized this categorization according to practice time, which seems to be a more reliable metric for future inferences.

Another extremely important factor that should be highlighted in epidemiological studies is the injury mechanisms. Nery et al. 8 subdivide them into two groups: atraumatic and traumatic injuries. Although simple, this division helps to understand the behavior of the injury process in the athlete. As presented by Bittencourt et al., 29 injuries are composed of a multimodal network with factors, of greater or lesser impact, which are connected. As a result, the precise identification of the mechanisms of injuries and how they occur is of paramount importance for the clinical practice. More accurate studies in this regard are needed, as reported in Hinz et al. 7

A consensus on the definition of injury in soccer stressed the importance of identifying the time between injury and return to full participation in the sport. 30 In the studies included 4 7 8 9 16 17 18 in the present review, only one 7 showed this type of assessment of great importance to also understand the severity of the injury. Only in the studies by Nicolini et al. 4 and Hinz et al., 7 the authors pointed out the impact of these injuries through the treatment received after the event.

The present systematic review indicates that future studies should be more categorical in their evaluations for better inference of results. To this end, it is necessary to include variables such as weekly training hours, time of return after the injury, and the mechanism of injury, for the promotion of improvements in class models, coping strategies, and mitigation of the risk of injury.

## Conclusion

There is a high prevalence of musculoskeletal injuries among jiu-jitsu practitioners. The most affected anatomical segments are the knee joint, chest, and rib region, followed by the shoulder joint. The related factors change according to certain variables, being more common during training in male individuals over 30 years of age and beginners in the sport.
