# Supplementary material for: Epidemiology of Injuries and Their Implications in Jiu-Jitsu Practitioners: An Integrative Systematic Review
Source: Rev Bras Ortop (Sao Paulo). 2024 Jun 22;59(3):e364–71. doi: 10.1055/s-0044-1785662 (PMC11193589; doi:10.1055/s-0044-1785662)
Supplement: Related texts

# Research article: Epidemiologia das lesões e suas implicações em praticantes de jiu-jitsu: Uma revisão sistemática integrativa

**Keywords:** artes marciais, esportes, medicina esportiva, transtornos traumáticos cumulativos

## Abstract

**Objetivo** Investigar a epidemiologia dos tipos de lesões entre praticantes de jiu-jitsu e sua incidência em diferentes níveis de habilidade e experiência por meio da questão: “Quais as características e a prevalência das lesões musculoesqueléticas em praticantes de jiu-jitsu?”

**Métodos** Desde o início do estudo, em agosto de 2020, foram pesquisados os bancos de dados MEDLINE, LILACS e SciELO. Foram incluídos estudos transversais, publicados entre 2018 e 2023, que investigaram a epidemiologia dos tipos de lesões ocorridas entre praticantes de jiu-jitsu e compararam sua incidência em diferentes níveis de habilidade e experiência. Para tanto, dois pesquisadores independentes realizaram a extração dos dados e avaliaram o risco de viés.

**Resultados** Sete estudos foram incluídos. Os resultados comuns envolveram 2.847 praticantes de jiu-jitsu. Houve uma alta prevalência de lesão na articulação do joelho e nas áreas do tórax e das costelas. Considerando a diferença de nível de experiência entre os praticantes, pôde-se observar que a maioria dos indivíduos incluídos eram iniciantes. Entre as faixas etárias observadas, homens acima de 30 anos de idade foram os que mais apresentaram lesões musculoesqueléticas, principalmente durante os treinos.

**Conclusão** Houve uma alta prevalência de lesões musculoesqueléticas entre os praticantes de jiu-jitsu. Os segmentos anatômicos mais acometidos foram a articulação do joelho, o tórax e a região das costelas, seguidos da articulação do ombro. Os fatores relacionados mudaram de acordo com algumas variáveis, sendo mais comuns durante o treinamento em indivíduos do sexo masculino com mais de 30 anos e iniciantes na modalidade.

## Introdução

As lutas em esportes de combate podem resultar em lesões de partes moles 1 e até mesmo traumatismos estruturais; 2 essas lesões podem ser graves e requerer artroplastia de determinadas estruturas, 3 além de poder levar à interrupção da luta ou ao afastamento da prática esportiva.

O jiu-jitsu é um esporte de combate em que os atletas utilizam técnicas de “finalização” sobre o adversário; muitas vezes, o resultado de tais técnicas é um bloqueio articular, denominado “chave de submissão” pelos praticantes. 4 A luta acontece com movimentos inesperados, rápidos, repetitivos e de alta sobrecarga para músculos e articulações. Associados ao treinamento de alto volume, esses fatores podem favorecer o aparecimento de lesões musculoesqueléticas e osteoarticulares. 5

Nessa arte marcial, o alto impacto físico e a exposição aos treinos diários corroboram os desfechos observados na literatura, com maior incidência de lesões durante os treinos do que durante as competições. 6 7 8 9

Alguns autores enfatizaram que a incidência de lesões é muito semelhante entre as artes marciais. Porém, há poucos estudos associados às lesões decorrentes especificamente da prática do jiu-jitsu. 4 5 10 11 Como as lesões são caracterizadas de acordo com a gravidade, o tempo de tratamento, a natureza e as características intrínsecas, 4 12 o conhecimento da incidência e da prevalência das lesões ortopédicas, bem como de suas características, gera benefícios diretos para o tratamento, prevenção e promoção de estratégias para tais eventos. Assim, o objetivo deste estudo foi investigar a epidemiologia dos tipos de lesões entre praticantes de jiu-jitsu, bem como a incidência em diferentes níveis de habilidade e experiência, por meio da questão: “Quais as características e a prevalência das lesões musculoesqueléticas entre praticantes de jiu-jitsu?”. Esta revisão sistemática sintetiza e traz evidências para a comunidade científica e clínica sobre essas condições e possível apoio à saúde dos praticantes de jiu-jitsu.

## Materiais e Métodos

## Estratégia de Busca

Esta revisão sistemática foi conduzida de acordo com as diretrizes da declaração Preferred Reporting Items for Systematic Reviews and Meta-Analyses (PRISMA) *.* 13 14 O protocolo do estudo foi elaborado e registrado no banco de dados Prospective Register of Systematic Reviews (PROSPERO) sob a identificação CRD42023422767. A busca foi realizada nas bases de dados eletrônicas MEDLINE (PubMed), LILACS e SciELO. A estratégia utilizada foi a combinação e adaptação dos descritores *medical subject headings* (MeSH), da National Library of Medicine: *jiu-jitsu* , *Brazilian jiu-jitsu* (“jiu-jitsu brasileiro”) e *injuries* (“lesões”) ( Tabela 1 ). A busca foi realizada desde o início do estudo até maio de 2023. As listas de referências bibliográficas foram revisadas para identificar outros possíveis estudos.

**Tabela 1:** Estratégias de busca

Busca	Pesquisa
#1	*(prevalence [MeSH Terms]* OR *prevalence* OR *prevalences* OR *occurrence* OR *frequency* OR *frequencies* OR *incidence* OR *epidemiology* OR *epidemiologic* OR *incidence* OR *incidences)* (“prevalência” [termos MeSH] OU “prevalência” OU “prevalências” OU “ocorrência” OU “frequência” OU “frequências” OU “incidência” OU “epidemiologia” OU “epidemiológica” OU “incidência” OU “incidências”)
#2	*(injuries* OR *injury* OR *sports injuries* OR *sports injury* OR *injuries, sports* OR *injury, sports* OR *injuries, athletic* OR *athletic injury* OR *injury, athletic)* (“lesões” OU “lesão” OU “lesões esportivas” OU “lesões esportivas” OU “lesões esportivas” OU “lesões esportivas” OU “lesões atléticas” OU “lesões atléticas” OU “lesões atléticas”)
#3	*(jiujitsu* OR *jiu-jitsu* OR *jujitsu* OR *Brazilian jiujitsu* OR *Brazilian jiu-jitsu)* (“jiujitsu” OU “jiu-jitsu” OU “jujitsu” OU “jiujitsu brasileiro” OU “jiu-jitsu brasileiro”)
#4	(#1 AND #2 AND #3) (#1 E #2 E #3)

A estratégia de População, Intervenção, Comparação, Desfechos e Estudos ( *Population* , *Intervention* , *Comparison* , *Outcomes* , *Studies* , PICOS, em inglês) foi utilizada para formular a questão nesta revisão sistemática, em que P representa os praticantes de jiu-jitsu, I e C não são aplicáveis, O é a prevalência de lesões musculoesqueléticas e os tipos mais frequentes entre os praticantes, e S representa os estudos observacionais. Esta revisão sistemática não inclui intervenção ou comparação.

## Seleção de Estudos

Os estudos identificados na busca foram inseridos em uma planilha do programa Excel (Microsoft Corp., Redmond, WA, Estados Unidos) para a exclusão de duplicatas. Dois pesquisadores independentes (HHPS e SPN) empregaram a estratégia de seleção baseada no conteúdo do título e do resumo. Posteriormente, os textos completos dos estudos foram recuperados para a avaliação de elegibilidade. Os critérios de inclusão foram estudos transversais que envolviam praticantes de jiu-jitsu e lesões musculoesqueléticas. Foram excluídos estudos publicados em idiomas que não inglês ou português e textos incompletos. Divergências entre os pesquisadores foram resolvidas por meio de discussões. Em caso de persistência de qualquer divergência, a avaliação de um terceiro pesquisador (CEG) foi solicitada.

## Extração de Dados e Avaliação do Risco de Viés

Dois pesquisadores independentes (HHPS e SPN) realizaram a extração de dados e avaliaram o risco de viés. A coleta de dados foi feita em um formulário predefinido, que abrangia o primeiro autor, o ano de publicação, o nível dos praticantes, a idade, o sexo, o número de atletas, a frequência de treinamento, a região anatômica da lesão musculoesquelética e o momento da lesão. O risco de viés em estudos observacionais de exposição foi avaliado por meio da ferramenta Risk of Bias In Non-Randomized Studies – of Exposure (ROBINS-E), da Cochrane Collaboration, 15 que abrange sete domínios metodológicos: confusão, mensuração da exposição, seleção de participantes, intervenções pós-exposição, dados faltantes, medida de resultados e seleção do resultado relatado. A pontuação é estabelecida como risco de viés baixo (-), alto (+) e incerto (?).

## Resultados

Na busca sistemática nas principais bases de dados eletrônicas, foram encontrados 34 artigos. Não foram selecionados estudos da literatura cinzenta, pois os registros identificados já constavam nas demais bases de dados. Após exclusão das duplicatas e conclusão da triagem de títulos e resumos, restaram 10 registros. Posteriormente, um registro foi descartado, e nove estudos foram considerados elegíveis para serem avaliados na íntegra. Depois da leitura do texto completo, sete estudos 4 7 8 9 16 17 18 foram finalmente incluídos para a realização da síntese qualitativa e quantitativa. A Fig. 1 mostra uma visão geral do processo de seleção.

**Fig. 1:** O diagrama de fluxo do processo de seleção dos estudos segundo a declaração Preferred Reporting Items for Systematic Reviews and Meta-Analyses (PRISMA).

## Características do Estudo

Os estudos transversais incluídos nesta revisão sistemática, 4 7 8 9 16 17 18 publicados entre 2018 e 2023, totalizaram 2.847 praticantes de jiu-jitsu. A Tabela 2 apresenta as características dos artigos incluídos.

**Tabela 2:** Características dos estudos que atenderam aos critérios de inclusão

Autores e ano de publicação		País	n	Objetivos	Coleta de dados
da Silva Junior et al., 16 2018		Brasil	108	Verificar as regiões do corpo afetadas, o sítio mais comum, o mecanismo e a gravidade das lesões em praticantes iniciantes e avançados de jiu-jitsu.	Questionário
Lopes et al., 17 2018		Brasil	31	Estudar as possíveis relações entre a prevalência de lesões e o sistema funcional de movimento de lutadores de jiu-jitsu.	Questionário
Petrisor et al., 18 2019		Canadá	70	Descrever as lesões vivenciadas durante o treinamento de jiu-jitsu, tanto nos treinos quanto nas competições, classificar o tipo de lesão e explorar as características do participante e da lesão associadas ao desejo de abandonar o jiu-jitsu após o evento.	Questionário
Moriarty et al., 9 2019		Estados Unidos	1.287	Determinar a taxa de incidência em seis meses e as lesões relacionadas ao padrão do jiu-jitsu e caracterizar associações entre lesões e nível de experiência, fatores demográficos e variáveis de treinamento.	Questionário
Nicolini et al., 4 2021		Brasil	96	Identificar o perfil epidemiológico das lesões ortopédicas presentes em praticantes de jiu-jitsu.	Questionário
Hinz et al., 7 2021		Alemanha	1.140	Quantificar a incidência de lesões relacionadas ao jiu-jitsu ao longo de três anos e detectar padrões comuns de lesões e fatores de risco entre os praticantes da modalidade.	Questionário
Nery et al., 8 2023		Brasil	115	Determinar a prevalência de lesões musculoesqueléticas em competidores de jiu-jitsu, bem como seu perfil e características.	Questionário

## Definição da Lesão Musculoesquelética no Esporte

As lesões musculoesqueléticas são comuns e representam um fardo significativo para o sistema de saúde. Segundo Caine et al., 19 Hoff e Martin 20 e Van Mechelen et al., 21 *lesões esportivas* é o nome coletivo para caracterizar todos os tipos de danos relacionados às atividades físicas. Corroborando a discussão dos autores, Timpka et al. 22 as definem etiologicamente como perda de funções corporais ou desvio de estruturas decorrente da transferência de energia durante a participação no esporte. Portanto, é possível distinguir lesões agudas, que ocorrem pela interação com uma força relativamente alta em curto tempo de estímulo, de lesões crônicas, causadas pelo efeito da força aplicada na repetição, mesmo em menor intensidade, como o uso excessivo, por exemplo. 23 24 Assim, com base nessa definição, esta revisão sistemática visa proporcionar compreensão sobre esses resultados da literatura.

## Epidemiologia da Lesão: Estrutura, Idade e Momento de Ocorrência

A distribuição da ocorrência de lesões musculoesqueléticas de acordo com os estudos incluídos 4 7 8 9 16 17 18 mostra alta prevalência delas na articulação do joelho (22%) e nas regiões do tórax e das costelas (22%), com taxas próximas à metade do total de casos relatados neste estudo. Considerando a diferença no nível de experiência entre os praticantes, é possível observar que a maioria dos indivíduos incluídos era das faixas branca e azul, ou seja, iniciantes na modalidade. A Fig. 2 mostra a distribuição total da prevalência de lesões e da experiência dos atletas.

**Fig. 2:** Características epidemiológicas das lesões e nível de experiência dos praticantes de jiu-jitsu.

Considerando as faixas etárias, foram incluídos participantes acima de 18 anos, o que dificulta a análise dos dados dos praticantes mais jovens. Nesse sentido, de acordo com os dados disponíveis, percebe-se que os praticantes do sexo masculino acima de 30 anos são mais suscetíveis a sofrer uma lesão musculoesquelética. Outra característica notável é a alta taxa de ocorrência de lesões durante os treinamentos em comparação com as competições. A Tabela 3 mostra os dados completos.

**Tabela 3:** Epidemiologia das características de pacientes com lesão

Estudo	Idade (anos)			Frequência das lesões (%)			Momento da lesão	
	Média ± (desvio padrão)	Inclusão	Maior frequência	Homens	Mulheres	Total	Competição	Treinamento
da Silva Junior et al. 16	28,3(±6,41)	Não relatada	Não relatada	Não relatado	Não relatada	100%	0%	100%
Lopes et al. 17	30,9(±7,3)	Não relatada	≥ 30	100%	Não relatada	100%	0%	100%
Petrisor et al. 18	–	> 18	≥ 30	Não relatada	Não relatada	91,4%	8,6%	100%
Moriarty et al. 9	29,5(±2,12)	> 18	26 a 35	15,5%	84,5%	59%	2%	98%
Nicolini et al. 4	27,6(±1,42)	18 a 45	Não relatada	86%	84%	84%	16%	100%
Hinz et al. 7	31,7(±7,9)	Não relatada	32,12	89,7	10,2%	48,7%	51,30%	48,70%
Nery et al. 8	25,8(±4,1)	Não relatada	Não relatada	Não relatada	Não relatada	85,2%	41%	59%

## Avaliação do Risco de Viés

De modo geral, houve um risco moderado de viés nos estudos incluídos 4 7 8 9 16 17 18 ( Fig. 3 ). As descrições dos protocolos e dos métodos abordados não foram apropriadas para uma interpretação completa dos fatores de confusão e medida dos desfechos. Participação, sequência aleatória e ocultação de alocação foram identificadas de forma robusta em três estudos. 4 8 9 Os estudos não deram muita ênfase à perda de dados ou à exposição errônea, bem como ao viés de medida dos desfechos. A Fig. 3 apresenta a avaliação de cada item de risco de viés dos estudos incluídos.

**Fig. 3:** Resumo dos julgamentos de revisão dos autores sobre cada item de risco de viés de cada estudo incluído.

## Discussão

O presente estudo é uma revisão sistemática da literatura sobre a prevalência de lesões musculoesqueléticas em praticantes de jiu-jitsu, com avaliação da relação entre os segmentos anatômicos mais acometidos, a idade, o sexo, a faixa e o tempo de lesão. Portanto, foi possível destacar as regiões anatômicas com maior incidência de lesões que mais deveriam chamar a atenção dos praticantes e instrutores de jiu-jitsu.

É importante ressaltar que as artes marciais são baseadas em princípios biomecânicos que podem causar graves danos aos praticantes. 5 25 Devido à técnica de luta que expõe os praticantes a quedas e choques com grande aplicação de força, as lesões ortopédicas acabam se tornando muito comuns na prática do esporte. Aproximadamente 90% dos atletas participantes relataram a ocorrência de pelo menos uma lesão. 26 Porém, ao examinar a incidência dessas lesões segundo variáveis demográficas, não foi possível encontrar um fator consistente que tivesse impacto significativo em sua ocorrência.

Esta revisão sistemática observou maior prevalência de lesões no joelho, tórax e tornozelo em comparação a outros segmentos anatômicos. Essa situação também se aplica ao avaliarmos estudos sobre outras artes marciais, como o judô. 27 Além disso, como observado por Pocecco et al., 28 dedos e mãos são os sítios mais acometidos por lesões no judô, diferentemente do jiu-jitsu, como mostram os dados do presente estudo. Em outras modalidades, como as artes marciais mistas, que permitem contato mais agudo e incisivo, como socos e chutes, a prevalência de lesões é maior na cabeça e no pescoço. Nessas modalidades, as lacerações, escoriações e contusões são as mais frequentes, além de condições como concussão.

Nesta revisão, evidenciou-se o baixo número de estudos sobre o tema, o que dificulta ainda mais a interpretação dos resultados. Fatores como a falta de padronização para considerar atletas iniciantes ou experientes também dificultaram a realização de inferências sobre os resultados. Para ilustrar, da Silva Junior et al. 16 consideraram atletas iniciantes aqueles nas faixas branca ou azul, ao passo que Petrisor et al. 18 consideraram atletas iniciantes apenas aqueles de faixa branca, sendo os demais atletas considerados avançados. Seria interessante que estudos futuros padronizassem essa categorização de acordo com o tempo de prática, o que parece ser uma métrica mais confiável para inferências futuras.

Outro fator de extrema importância que deve ser destacado nos estudos epidemiológicos são os mecanismos de lesão. Nery et al. 8 os subdividem em dois grupos: lesões atraumáticas e traumáticas. Apesar de simples, essa divisão ajuda a compreender o comportamento do processo da lesão no atleta. Segundo Bittencourt et al., 29 a lesão é composta por uma rede multimodal com fatores interligados de maior ou menor impacto. Por isso, identificar com precisão os mecanismos das lesões e como elas ocorrem é de suma importância para a prática clínica. Estudos mais precisos a esse respeito são necessários, como observado por Hinz et al. 7

Um consenso sobre a definição de lesão no futebol ressaltou a importância de identificar o tempo entre a lesão e o retorno à participação plena no esporte. 30 Nos estudos incluídos 4 7 8 9 16 17 18 em nesta revisão, apenas um 7 fez essa avaliação de grande importância para também compreender a gravidade da lesão. Somente os estudos de Nicolini et al. 4 e Hinz et al. 7 apontaram o impacto dessas lesões por meio do tratamento recebido após o evento.

Esta revisão sistemática indica que estudos futuros devem ser mais categóricos em suas avaliações para possibilitar uma melhor inferência dos resultados. Para tanto, é necessário incluir variáveis como horas semanais de treino, tempo de retorno após a lesão e mecanismo de lesão, para a promoção de melhoras nos modelos de aula, nas estratégias de enfrentamento e na mitigação do risco de lesão.

## Conclusão

Houve uma alta prevalência de lesões musculoesqueléticas entre os praticantes de jiu-jitsu. Os segmentos anatômicos mais acometidos foram a articulação do joelho, o tórax e a região das costelas, seguidos da articulação do ombro. Os fatores relacionados mudaram de acordo com determinadas variáveis, sendo mais comuns durante o treinamento em indivíduos do sexo masculino com mais de 30 anos e iniciantes na modalidade.
